# Supplementary material for: High frequency acoustic cell stimulation promotes exosome generation regulated by a calcium-dependent mechanism
Source: Commun Biol. 2020 Oct 5;3:553. doi: 10.1038/s42003-020-01277-6 (PMC7536404; doi:10.1038/s42003-020-01277-6)
Supplement: Supplementary file 1 — Supplementary Information [file 42003_2020_1277_MOESM1_ESM.pdf]

Supplementary Information

# High Frequency Acoustic Cell Stimulation Promotes Exosome Generation Regulated by a Calcium-Dependent Mechanism

Lizebona August Ambattu,[1] Shwathy Ramesan,[1] Chaitali Dekiwadia,[2] Eric Hanssen,[3] Haiyan Li,[4] and Leslie Y. Yeo[1,*]

[1] Micro/Nanophysics Research Laboratory, School of Engineering, RMIT University, Melbourne, VIC 3000, Australia
[2] RMIT Microscopy and Microanalysis Facility, RMIT University, Melbourne, VIC 3000, Australia
[3] Advanced Microscopy Facility, Bio21 Molecular Science and Biotechnology Institute, University of Melbourne, Melbourne, 3010, Australia
[4] School of Biomedical Engineering & Med-X Research Institute, Shanghai Jiao Tong University, Shanghai 200030, China
*E-mail: leslie.yeo@rmit.edu.au

## Supplementary Figures

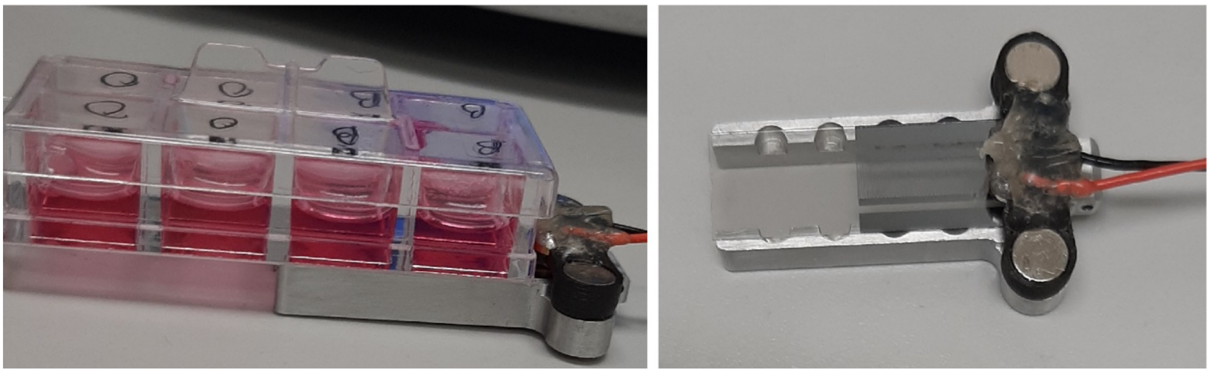

**Supplementary Figure 1** Experimental setup (left), which comprises a glass-bottom culture plate containing the cells mounted atop the SRBW device (also shown in top view on the right). A layer of silicon oil is placed in between the device and the glass-bottom culture plate, although this is too thin to be seen.

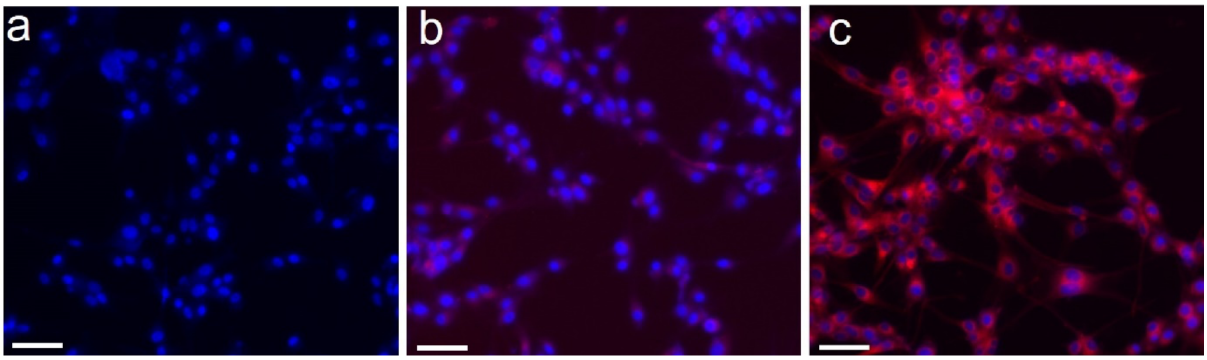


**Supplementary Figure 2** Microscopy images showing the internalisation of exosomes tagged with BODIPY$^{\text{TM}}$ TR ceramide in cells counterstained with Hoechst 33342 after **a** 1 hr, **b** 4 hrs and **c** 18 hrs of incubation. The scale bars denote a length of 50 µm. Although exosome uptake in the cells can first be seen after 4 hrs, prolonged incubation led to more significant internalisation, suggesting that overnight post-exposure incubation results in a decrease in the overall exosome quantity.

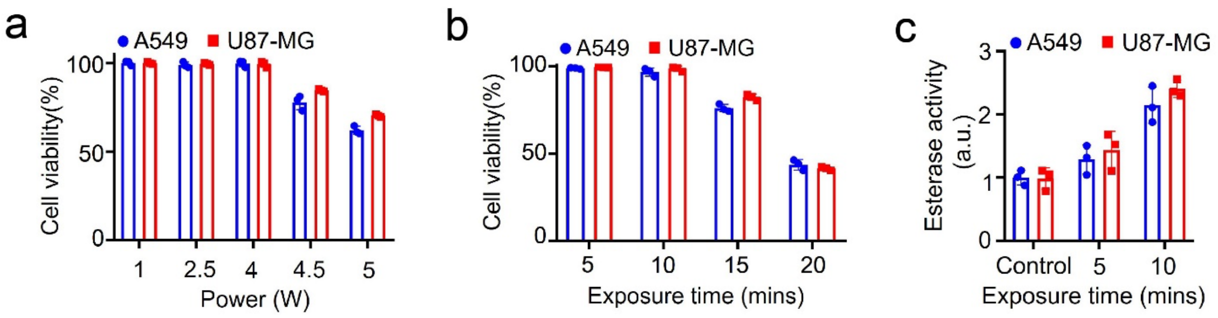


**Supplementary Figure 3** Cell viability following acoustic irradiation at various **a** input powers and **b** exposure times; in the former, the exposure time is fixed at 10 mins, whereas in the latter, the power is fixed at 4 W. **c** Relative esterase activity following SRBW exposure at 4 W for 5 and 10 mins compared to that of the unexposed control. The data are represented in terms of the mean value ± the standard error over triplicate runs.

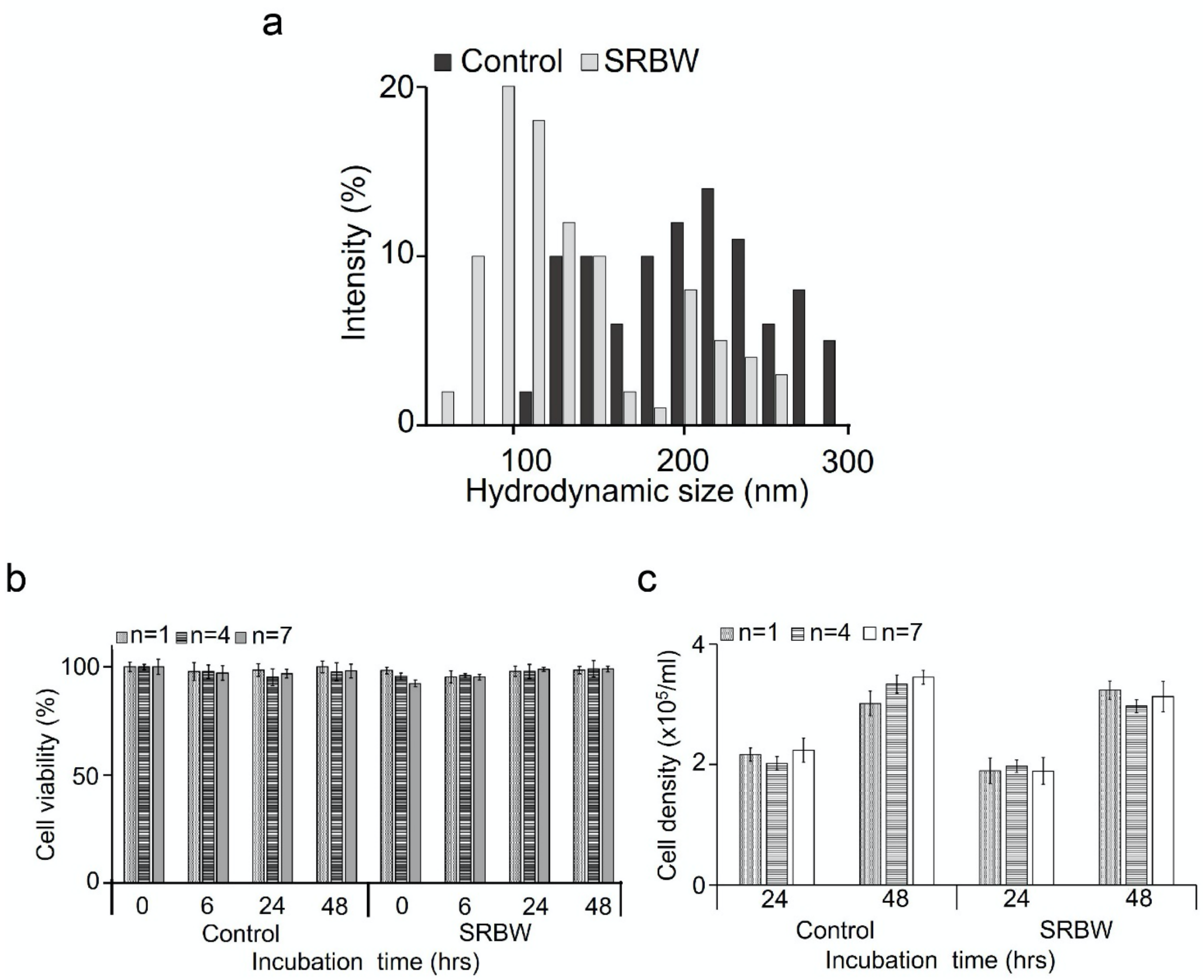


**Supplementary Figure 4** **a** Hydrodynamic size of the exosomes isolated from A549 cells. **b** A549 cell viability, as measured from an MTT assay, and, **c** A549 cell population density as a function after successive number *n* of excitation–incubation cycles. The data are represented in terms of the mean value ± the standard error over triplicate runs.

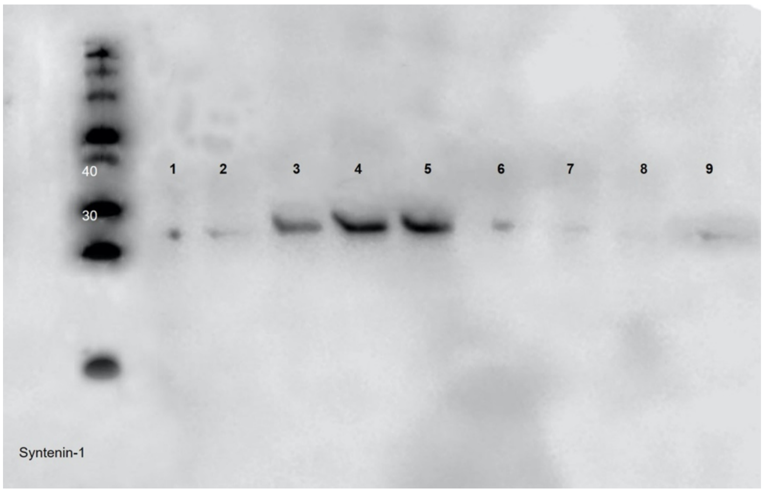


**Supplementary Figure 5** Full scan of the Western blot data (syntenin-1) of exosomes isolated from A549 cells. Lanes 1, 2 and 3 pertain to the exosome lysate from SRBW treated cells after successive excitation–incubation cycles n = 1, n = 4 and n = 7, respectively, and lanes 6, 7, and 8 pertain to the exosome lysate from the control (untreated) cells at cycles n = 7, n = 4 and n = 1, respectively. Lanes 5 and 9 pertain to the exosome lysate of SRBW treated and control (untreated cells), respectively from which the data in Figure 2d was spliced.

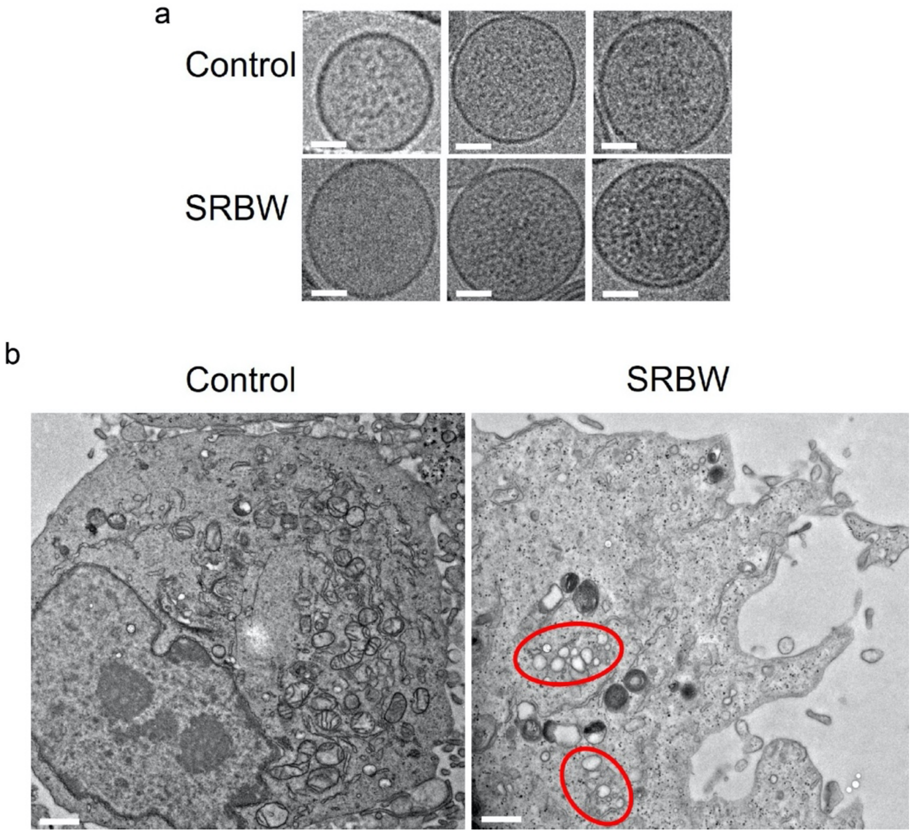


**Supplementary Figure 6 a** Representative cryo-EM images of the exosomes isolated from U87-MG cells; the scale bars represent a length of 50 nm. **b** TEM images showing significantly more MVBs (circled in red) in U87-MG cells that were irradiated with the SRBW (right) compared to that in the control (left); the scale bars represent a length of 1 µm.
