# Supplementary material for: High frequency acoustic cell stimulation promotes exosome generation regulated by a calcium-dependent mechanism
Source: Commun Biol. 2020 Oct 5;3:553. doi: 10.1038/s42003-020-01277-6 (PMC7536404; doi:10.1038/s42003-020-01277-6)
Supplement: Supplementary file 3 — Reporting Summary [file 42003_2020_1277_MOESM3_ESM.pdf]

nature research

Corresponding author(s): Leslie Y Yeo

Last updated by author(s): Aug 21, 2020

Reporting Summary

Nature Research wishes to improve the reproducibility of the work that we publish. This form provides structure for consistency and transparency in reporting. For further information on Nature Research policies, see our Editorial Policies and the Editorial Policy Checklist.

Statistics

For all statistical analyses, confirm that the following items are present in the figure legend, table legend, main text, or Methods section.

n/a	Confirmed	
☐	☒	The exact sample size (n) for each experimental group/condition, given as a discrete number and unit of measurement
☐	☒	A statement on whether measurements were taken from distinct samples or whether the same sample was measured repeatedly
☐	☒	The statistical test(s) used AND whether they are one- or two-sided<br>*Only common tests should be described solely by name; describe more complex techniques in the Methods section.*
☐	☒	A description of all covariates tested
☒	☐	A description of any assumptions or corrections, such as tests of normality and adjustment for multiple comparisons
☐	☒	A full description of the statistical parameters including central tendency (e.g. means) or other basic estimates (e.g. regression coefficient) AND variation (e.g. standard deviation) or associated estimates of uncertainty (e.g. confidence intervals)
☐	☒	For null hypothesis testing, the test statistic (e.g. F, t, r) with confidence intervals, effect sizes, degrees of freedom and P value noted<br>*Give P values as exact values whenever suitable.*
☒	☐	For Bayesian analysis, information on the choice of priors and Markov chain Monte Carlo settings
☒	☐	For hierarchical and complex designs, identification of the appropriate level for tests and full reporting of outcomes
☒	☐	Estimates of effect sizes (e.g. Cohen's d, Pearson's r), indicating how they were calculated

Our web collection on statistics for biologists contains articles on many of the points above.

Software and code

Policy information about availability of computer code

Data collection	Not applicable
Data analysis	Not applicable

For manuscripts utilizing custom algorithms or software that are central to the research but not yet described in published literature, software must be made available to editors and reviewers. We strongly encourage code deposition in a community repository (e.g. GitHub). See the Nature Research guidelines for submitting code & software for further information.

Data

Policy information about availability of data

All manuscripts must include a data availability statement. This statement should provide the following information, where applicable:

- Accession codes, unique identifiers, or web links for publicly available datasets
- A list of figures that have associated raw data
- A description of any restrictions on data availability

The data that support the findings of this study are available from the corresponding author upon reasonable request.

Field-specific reporting

Life sciences study design

All studies must disclose on these points even when the disclosure is negative.

Sample size	Not applicable
Data exclusions	No data was excluded from the analysis
Replication	All experiments were performed at least in triplicate
Randomization	Not applicable
Blinding	Not applicable

Reporting for specific materials, systems and methods

We require information from authors about some types of materials, experimental systems and methods used in many studies. Here, indicate whether each material, system or method listed is relevant to your study. If you are not sure if a list item applies to your research, read the appropriate section before selecting a response.

Materials & experimental systems

n/a	Involved in the study	
☐	☒	Antibodies
☐	☒	Eukaryotic cell lines
☒	☐	Palaeontology and archaeology
☒	☐	Animals and other organisms
☒	☐	Human research participants
☒	☐	Clinical data
☒	☐	Dual use research of concern

Methods

n/a	Involved in the study	
☒	☐	ChIP-seq
☒	☐	Flow cytometry
☒	☐	MRI-based neuroimaging

Antibodies

Antibodies used

anti-GAPDH mouse antibody (Cell Signaling Technology Inc. (Danvers, MA, USA);2118S;14C10;14)
anti-ALIX mouse antibody (Cell Signaling Technology Inc. (Danvers, MA, USA);2171S;3A9;5)
anti-Rab 27a rabbit antibody (Cell Signaling Technology Inc. (Danvers, MA, USA);69295S;1)
anti-TSG101 mouse antibody (Thermo Fisher Scientific Pty. Ltd. (Scoresby, VIC, Australia);MA1-23296;UE2767101G)
anti-syntenin-1 rabbit antibody (Thermo Fisher Scientific Pty. Ltd. (Scoresby, VIC, Australia);PA5-28826;UF2795833)
anti-calnexin rabbit antibody (Abcam (Cambridge, UK);AB22595;GR3276815-2)
anti-flotillin-1 mouse antibody (BD Biosciences (San Jose, CA, USA);610820;9010889)
anti-CD63 mouse antibody (Invitrogen (Carlsbad, CA, USA);10628D;RSP-PF180117T2)
antimouse HRP conjugated antibody (Cell Signaling Technology Inc. (Danvers, MA, USA);7075P5;36)
anti-rabbit HRP-conjugated antibody (Cell Signaling Technology Inc. (Danvers, MA, USA),7074S;25)
Biotinylated Protein Ladder Detection Pack (Cell Signaling Technology Inc. (Danvers, MA, USA),7727;26)
Precision Plus Protein™ WesternC™ Blotting Standards (BIO-RAD Laboratories (New South Wales, Australia):1610376;L001652 A)

Validation

The manuscript contains Western blot data with the following antibodies:
anti-GAPDH mouse antibody
anti-ALIX mouse antibody
anti-Rab 27a rabbit antibody
anti-TSG101 mouse antibody
anti-syntenin-1 rabbit antibody
anti-calnexin rabbit antibody
anti-flotillin-1 mouse antibody
anti-CD63 mouse antibody
antimouse HRP conjugated antibody
anti-rabbit HRP-conjugated antibody
Biotinylated Protein Ladder Detection Pack
Precision Plus Protein™ WesternC™ Blotting Standards
All antibodies were validated by the suppliers for Western blotting.

Eukaryotic cell lines

Policy information about cell lines

Cell line source(s)	ATCC
Authentication	A morphology check by microscope and Mycoplasma detection was carried out, in line with the recommended tests by ATCC
Mycoplasma contamination	The cell line used tested negative for Mycoplasma contamination
Commonly misidentified lines (See ICLAC register)	Not applicable
